# Association of patellofemoral osteoarthritis on patient-reported outcomes after medial unicompartmental knee arthroplasty: a retrospective cohort study

**DOI:** 10.2340/17453674.2024.42575

**Published:** 2025-01-09

**Authors:** Jonathan Winther OLSEN, Christian Bredgaard JENSEN, Kristine Ifigenia BUNYOZ, Anders Flygenring BAGGE, Kirill GROMOV, Anders TROELSEN

**Affiliations:** Clinical Orthopaedic Research Hvidovre, Department of Orthopaedic Surgery, Copenhagen University Hospital Hvidovre, Hvidovre, Denmark

## Abstract

**Background and purpose:**

In contemporary medial unicompartmental knee arthroplasty (mUKA), non-lateral patellofemoral osteoarthritis (PFOA) is not considered a contraindication. However, we still lack knowledge on the association of PFOA severity on patient reported outcome measures (PROMs) after mUKA. We aimed to examine the association between PFOA severity and PROM-score changes after mUKA.

**Methods:**

We included 549 mobile-bearing mUKAs. PFOA was graded intraoperatively as 0 = normal cartilage, 1–2 = superficial changes or < 50% of depth, and 3–4 = changes of > 50% of depth or to the bone, using the International Cartilage Repair Society (ICRS) cartilage lesion classification system. All patients completed the Oxford Knee Score (OKS), Activity and Participation Questionnaire (APQ), and Forgotten Joint Score (FJS), preoperatively and 3, 12, and 24 months postoperatively. PROM changes were compared using linear regression models adjusted for sex, age, body mass index, and preoperative PROM score.

**Results:**

We found no significant differences in OKS, FJS, and APQ change when comparing group 3–4 with group 0 at any follow-up. When comparing group 1–2 with 0 we found a statistical but not clinical significantly higher change in OKS scores at 24-month follow-up (2.5, 95% confidence interval [CI] 0.36–4.6) and in APQ scores at 24-month follow-up (10.6, CI 1.2–20.0) in favor of group 1–2.

**Conclusion:**

Severe PFOA, excluding severe lateral facet PFOA, had no negative association on PROM score development following mobile-bearing mUKA.

Medial unicompartmental knee arthroplasty (UKA) is a surgical treatment for anteromedial knee osteoarthritis (OA) [1]. The indications for medial UKA have expanded over the past 2 decades, and its relative use in primary osteoarthritis treatments has increased [2]. UKA has fewer complications, quicker recovery, more same-day surgery, better early patient-reported outcome measures (PROMs), and is more cost-effective than total knee arthroplasty (TKA) [3-7].

In the past, patellofemoral osteoarthritis (PFOA) was considered a contraindication to unicompartmental knee arthroplasty (UKA) [8]. However, more recent studies have indicated that wear in the trochlear groove did not have a negative impact on PROM scores [9-12] or revision rates [9,10]. A recent meta-analysis found no significant differences in functional outcomes between patients with and without PFOA [13]. However, despite current knowledge, there is still a lack of insight into whether patients PFOA have different short-term developments in PROM scores, which is why further research is still relevant [13].

The primary aim of our study is to examine the association between the degree of PFOA and PROM scores at 3, 12, and 24 months after medial UKA.

## Methods

### Study design and participants

This retrospective cohort study included 549 patients treated with medial UKA between February 1, 2016, and December 31, 2020, at 1 university hospital. The study compares PROM scores between 3 groups of patients with increasing PFOA.

Due to the parallel-group multi-arm design of this cohort study, the reporting of this study adheres to the Consolidated Standards Of Reporting Trials (CONSORT) and the multi reporting of multi-arm parallel-group extension [14].

### Indication and surgery

Surgeons evaluated indications for medial UKA using standard anteroposterior and lateral knee radiographs with supplemental skyline and stress radiographs if deemed necessary. UKA was performed in patients with anteromedial OA following validated and commonly accepted criteria [15]. Inflammatory arthritis, posttraumatic arthritis, and severe lateral facet PFOA were considered contraindications. Patients who underwent surgery contralaterally were included as 2 separate UKA cases, each with associated PROM scores. The surgeries were performed using a standard minimally invasive technique using an anteromedial skin incision and an arthrotomy extending from the vastus medialis to an inch below the joint line. The exposure was sufficient for full inspection of the trochlear groove. All the surgeries were performed in a fast-track setup, which has previously been described [16]. All the surgeries were performed using the uncemented mobile-bearing Oxford medial unicompartmental prothesis (Zimmer Biomet, Warsaw, IN, USA).

### Grading of PFOA

An intraoperative assessment of the status of the trochlear grove was collected for each knee by the surgeon; however, they did not specify the location of the PFOA but reported the most severe grade observed. The surgeons used the International Cartilage Repair Society (ICRS) cartilage lesion classification system [17] to grade the trochlear wear intraoperatively. The surface was graded using a 5-point scale: normal (grade 0), nearly normal/superficial lesions (grade 1), abnormal/lesions extending down to < 50% of cartilage depth (grade 2), severely abnormal/ cartilage defects extending down to > 50% of cartilage depth (grade 3), and severely abnormal/defect extending through the subchondral bone (grade 4). For data analysis purposes grades were grouped into 3 classes: grade 0, grades 1–2, and grades 3–4.

### Outcomes

The PROM’s included the Oxford Knee Score (OKS), Activity and Participation Questionnaire (APQ), and Forgotten Joint Score (FJS), which are all validated for patients having a knee arthroplasty [18-21]. The OKS ranges from 0–48 (worst to best), whereas the APQ and FJS both range from 0–100 (worst to best). The OKS is a 12-item PROM assessing both pain and physical function, where standardized answer options are given (5-point Likert scale) and each question is assigned a score from 0 to 4. APQ consists of 8 questions where each question is ranges from “strongly disagree” to “strongly agree” with a total of 4 subclassifications giving a number from 1–4 that assess participation in usual daily activities. FJS is a scoring system containing 12 questions that analyze the patient’s ability to forget about a potentially painful joint. All 3 questionnaires were completed preoperatively and at 3, 12, and 24 months after surgery. To increase response rate, the forms were sent once by e-mail and later once by letter in the case of incomplete or missing reply. The change in PROM scores for each individual patient was calculated by subtracting the preoperative score from the postoperative scores at each follow-up.

We calculated the proportion of patients reaching the patient acceptable symptom state (PASS), indicating the proportion of UKAs with an acceptable postoperative outcome after 12 months. The threshold value for OKS (PASS-OKS) was set to 32.7 [22]. We also calculated the proportion of patients achieving the minimal important change (MIC), reflecting the proportion of patients within each group who experienced a clinically significant improvement after 12 months. The threshold value for OKS (MIC-OKS) was set to 7.1 [22]. The thresholds for PASS and MIC in OKS were based on a study from a similar UKA population at the same university hospital treated by the same surgeons; however, no cohort specific PASS or MIC for FJS and APQ has currently been computed.

### Statistics

To address missing data in body mass index (BMI), trochlear wear classification, and PROM scores, we performed multiple imputation using the Multivariate Imputation by Chained Equations (MICE) method, specifically applying predictive mean matching (PMM) for continuous variables and logistic or polynomial regression for categorical variables. Variables subjected to imputation were BMI, trochlear wear classification, OKS, FJS, and APQ, preoperatively and at 3 months’, 12 months’, and 24 months’ follow-up. Age, sex, BMI, American Society of Anesthesiologists Classification (ASA) score, PROM scores and trochlear wear classification were included in the imputation model. We conducted a total of 5 imputations with 50 iterations per imputation. Data presented after imputation is presented as pooled means and standard deviations (SD) or ranges of minimum and maximum in categorical variables (both in absolute numbers and percentages) for all 5 imputations.

Before imputation, BMI was missing in 12 (2.2%) patients, trochlear wear classification was missing in 24 (4.4%), and PROM scores were missing in between 10% and 19% of patients depending on the follow-up time and specific score (Tables 1 and 2, see Appendix). Patient characteristics and preoperative PROM scores in the imputed cohort are reported in Table 3. Patient characteristics and PROM scores for the complete-case (non-imputed) cohort of 440 mUKA patients with complete data in trochlear wear and preoperative PROM scores are reported in Table 4 (see Appendix).

**Table 1:** Patient characteristics before imputation (N = 549). Values are count (%) unless otherwise specified

Age, mean (SD)	66.7 (9.5)
Female sex	290 (53)
ASA 1–2	451 (82)
BMI, mean (SD)	30.3 (5.8)
Missing	12 (2.2)
Trochlea wear grade	
0	191 (35)
1–2	250 (46)
3–4	84 (15)
Missing	24 (4.4)

BMI = body mass index. SD = standard deviation.

**Table 2:** Preoperative and follow-up scores before imputation. Values are mean (SD) unless otherwise specified

Factor	OKS	FJS	APQ
Preoperative score	23.4 (7.5)	18.1 (16)	14.3 (17)
Missing, n (%)	80 (15)	89 (16)	87 (16)
3-month scores	33.9 (8.6)	52.4 (25)	46.7 (30)
Missing, n (%)	59 (11)	59 (11)	57 (10)
12-month score	38.2 (8.5)	61.8 (27)	59.4 (32)
Missing, n (%)	81 (15)	81 (15)	80 (15)
24-month score **^a^**	39.1 (8.5)	64.4 (28)	61.9 (34)
Missing, n (%)	82 (19)	83 (19)	82 (19)

a At 24 months only patients that were operated on at least 2 years before data collection were included (n = 427).

For abbreviations, see Table 3.

**Table 3:** Patients characteristics after imputation. Values are mean (SD) unless otherwise specified

Factor	Total (n = 549)	Trochlear wear		
		0 (n = 200–204)	1–2 (n = 256–262)	3–4 (n = 85–91)
Age	66.7 (9.5)	65.9 (9.7)	67.1 (9.6)	67.6 (8.8)
Female sex, n (%)	290 (53)	107–111 (54–55)	142–145 (55–55)	35–40 (49–53)
ASA 1–2, n (%)	451 (82)	165–169 (82–84)	213–219 (83–84)	64–70 (75–78)
BMI	30.3 (5.8)	29.5 (5.8)	30.5 (5.6)	31.4 (6.3)
Preoperative scores				
OKS	23.3 (7.5)	23.9 (7.8)	23.1 (7.0)	22.7 (8.1)
FJS	18.1 (16)	18.5 (17)	17.5 (15)	18.8 (15)
APQ	14.2 (17)	14.4 (19)	13.5 (16)	15.9 (18)

BMI = body mass index, IQR = interquartile range, OKS = Oxford Knee Score,

APQ = Activity and Participation Questionnaire, FJS = Forgotten Joint Score,

SD = standard deviation.

Missing data for trochlear wear group, BMI, and preoperative PROM scores (OKS, APQ, FJS) were imputed, see Statistics. The characteristics of the complete-case (non-imputed) cohort are reported in Table 4 (see Appendix).

**Table 4:** Patients characteristics in complete-case cohort

Factor	Total (n = 440)	Trochlear wear		
		0 (n = 158)	1–2 (n = 211)	3–4 (n = 71)
Age, mean (SD)	66.6 (9.3)	65.8 (9.6)	67.1 (9.4)	67.1 (8.4)
Female sex, n (%)	235 (53)	86 (54)	121 (57)	28 (39)
ASA 1–2, n (%)	367 (83)	133 (84)	176 (83)	58 (82)
BMI, mean (SD)	30.4 (5.8)	29.8 (5.9)	30.6 (5.7)	31.1 (5.8)
missing	10	6	3	1
Preoperative scores, mean (SD)				
OKS	23.4 (7.6)	24.0 (8.0)	23.1 (7.0)	22.8 (8.2)
FJS	18.0 (16)	18.7 (17)	17.3 (15)	18.6 (15)
APQ **^a^**	6 (3 to 22)	6 (0–22)	9 (3–19)	9 (3–27)

a Values are median (IQR). For abbreviations, see Table 3.

Distribution of data was evaluated using histograms and quantile–quantile plots. Normally distributed data is presented with means and standard deviations (SD) and non-normal data is presented with medians and interquartile range (IQR). PROM score changes at each follow-up were compared between each trochlear wear classification group using pooled linear regression models across all 5 imputations, both crude and adjusted for the potential confounders: sex, age, BMI, and preoperative PROM scores. The proportion of patients achieving PASS and MIC was compared between trochlear wear classification groups using pooled logistic regression models across all 5 imputations, both crude and adjusted for the potential confounders: sex, age, BMI, and preoperative PROM scores. As a sensitivity analysis, and to evaluate the association of imputation, all statistical tests were also applied to the complete-case (non-imputed) cohort of 440 mUKA patients with complete data in trochlear wear and preoperative PROM scores . No adjustments for multiplicity were applied.

R version 4.3.0 (R Core Team, 2023; R Foundation for Statistical Computing, Vienna, Austria) was used for the statistical analysis together with R Studio version 2024.4.2 (Posit team, 2024) and imputation was conducted using the MICE package version 3.16.0.

### Ethics, funding, data sharing plan, and disclosures

In Denmark, observational register-based studies using questionnaire data require no approval from the national research ethical committee. The PROM questionnaire register was approved by the Knowledge Centre on Data Protection Compliance, Capital Region of Denmark (P-2022-290). The local ethics committee has approved the access to chart data used in this study under the authority of the hospital board of directors.

This research did not receive any specific grant from funding agencies in the public, commercial, or not-for-profit sectors. CBJ has received PhD funding from a grant from the Novo Nordisk Foundation unrelated to this study. KG and AT have received research support and speaker fees from Zimmer Biomet, and AT has received research support from Pfizer. All the above conflicts are unrelated to this study. Complete disclosure of interest forms according to ICMJE are available on the article page, doi: 10.2340/17453674.2024.42575

## Results

**Figure 1:**
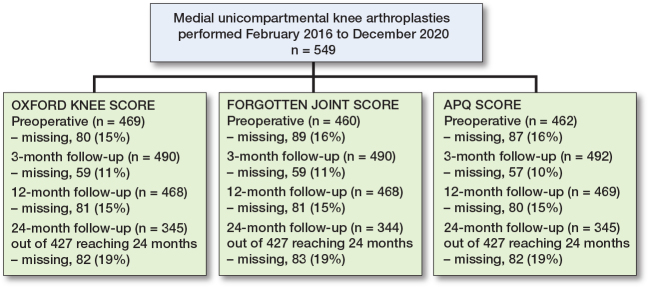
Patient flowchart. At 24-months’ follow-up at the time of data collection, only 427 patients had been operated on at least 24 months ago and were considered eligible for analysis at 24 months.

Figure 1 shows the included patients who had mUKA between February 1, 2016, and December 31, 2020. Patients might have missing data at 1 or more of the follow-ups. Of the 549 included patients, only 427 were operated on more than 2 years from data collection in April 2022, and only these 427 were included in analyses at 24 months. The mean time from surgery until questionnaire responses at the 3 follow-ups was: 3 months = 12.9 (SD 6.0) weeks, 12 months = 50.3 (SD 3.7) weeks, and 24 months = 100.9 (SD 4.7) weeks. If forms were still not available by April 2022, these were noted as missing.

Patient characteristics subdivided by the degree of trochlear wear are presented in Table 3. Mean age was 67 (range 29–93) years and mean BMI was 30 (range 19.6–53.1). 53% of the patients were women (n = 290). We found no major differences in patient characteristics between patients with complete and incomplete postoperative questionnaires (data not shown).

**Figure 2:**
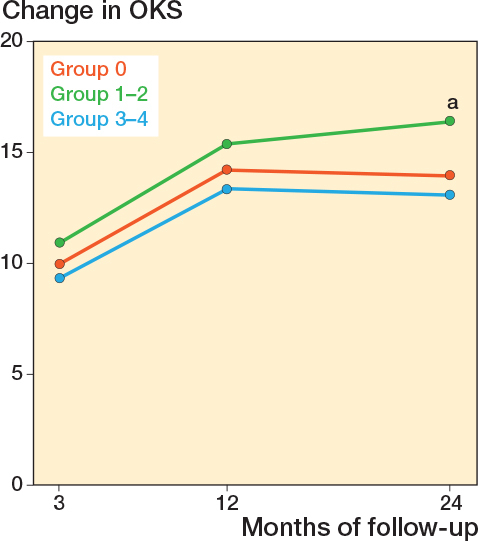
Change in Oxford Knee Score (OKS) from preoperative to 3-, 12-, and 24-month follow-up, for each of the trochlear wear groups. The change was calculated by subtracting the preoperative score from each follow-up score for the individual patient. **^a^** Indicates a significant difference between group 1–2 and group 0, at 24 months.

**Figure 3:**
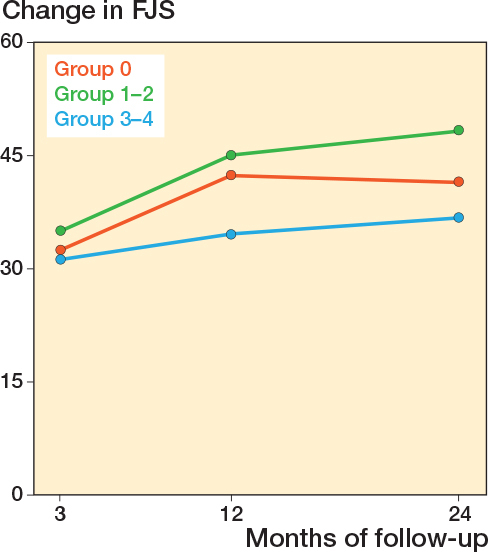
Change in Forgotten Joint Score (FJS) from preoperative to 3-, 12-, and 24-month follow-up, for each of the trochlear wear groups. Also see legend to Figure 2

**Figure 4:**
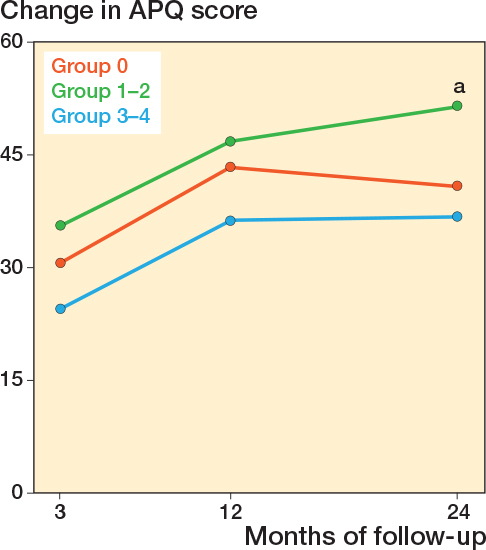
Change in Activity and Participation Questionnaire (APQ) score from preoperative to 3-, 12-, and 24-month follow-up, for each of the trochlear wear groups. Also see legend to Figure 2.

Differences in changes in PROM score between group 3-4 and group 0 did not reach significance. The largest estimate difference in OKS change at 12 months was –1.5 (CI –4.3 to 1.4). For FJS, the largest difference in change score at 12 months was –8.3 (CI –17 to 0.4). For APQ the larges difference in change score at 12 months was –7.2 (CI –18 to 3.2) (Table 5). When comparing group 1–2 with group 0, significant differences in PROM score changes were observed for OKS and APQ. For the OKS, we found a significantly larger change of 2.5 (CI 0.36–4.6) for group 1–2 compared with group 0 at 24-month follow-up. For the APQ, we found that group 1–2 had a significantly larger change compared with group 0 at 24-month follow-up (Table 5). In the analysis on the complete-case (non-imputed) data, group 1–2 also had a significantly larger change in APQ score at 3 months compared with group 0 (Table 6, see Appendix) Absolute chances in PROM scores for each follow-up are individually graphically illustrated (Figures 2-4).

**Table 5:** Change in PROM scores at 3-, 12- and 24-months’ follow-up

Follow-up Trochlear wear group	Mean (SD)	Estimated change	
		Crude (CI)	Adjusted (CI)
OKS at 3 months (n = 549)			
0	9.9 (9.0)		
1–2	10.9 (9.2)	0.95 (–0.87 to 2.8)	0.58 (–1.1 to 2.3)
3–4	9.3 (9.0)	–0.60 (–0.88 to 1.7)	–1.3 (–3.5 to 0.81)
OKS at 12 months (n = 549)			
0	14.1 (9.1)		
1–2	15.3 (9.0)	1.2 (–1.0 to 3.4)	0.80 (–1.3 to 2.9)
3–4	13.3 (9.5)	–0.86 (–4.0 to 2.3)	–1.5 (–4.3 to 1.4)
OKS at 24 months (n = 427) **^a^**			
0	13.9 (9.2)		
1–2	16.4 (8.6)	2.5 (0.36 to 4.6) **^b^**	2.4 (0.47 to 4.2) **^b^**
3–4	13.0 (10.4)	–0.87 (–4.4 to 2.6)	–1.4 (–4.7 to 1.8)
FJS at 3 months (n = 549)			
0	32.5 (26.8)		
1–2	35.1 (26.9)	2.5 (–2.7 to 7.8)	1.5 (–3.3 to 6.4)
3–4	31.3 (24.7)	–1.2 (–8.2 to 5.8)	–1.9 (–8.3 to 4.5)
FJS at 12 months (n = 549)			
0	42.4 (29.3)		
1–2	45.0 (28.5)	2.6 (–3.4 to 8.6)	1.6 (–4.0 to 7.3)
3–4	34.5 (26.8)	–7.8 (–17 to 1.4)	–8.3 (–17 to 0.37)
FJS at 24 months (n = 427) **^a^**			
0	41.5 (30.7)		
1–2	48.3 (28.6)	6.8 (–0.01 to 14)	6.7 (–0.60 to 13)
3–4	36.8 (30.4)	–4.7 (–15 to 5.2)	–4.2 (–14 to 5.3)
APQ at 3 months (n = 549)			
0	30.5 (30.1)		
1–2	35.5 (30.6)	5.0 (–0.68 to 11)	4.8 (–0.7 to 10)
3–4	24.5 (29.3)	–6.0 (–14 to 1.9)	–4.8 (–13 to 2.8)
APQ at 12 months (n = 549)			
0	43.4 (34.8)		
1–2	46.7 (33.6)	3.3 (–4.8 to 12)	3.2 (–4.3 to 11)
3–4	36.3 (35.6)	–7.2 (–18 to 3.2)	–4.5 (–14 to 4.8)
APQ at 24 months (n = 427) **^a^**			
0	40.8 (37.3)		
1–2	51.4 (34.4)	10.6 (1.2 to 20) **^b^**	11.5 (2.9 to 20) **^b^**
3–4	36.7 (39.0)	–4.2 (–19 to 10)	–1.6 (–15 to 12)

For abbreviations, see Table 1. CI = 95% confidence interval. To calculate the change scores, the preoperative score was subtracted at each subsequent follow-up.

Results are pooled multiple linear regression models applied to 5 imputed datasets, showing both the crude estimates and estimates adjusted for sex, BMI, age, and preoperative score. Trochlear wear 0 was used as reference group.

a At 24 months only the patients operated on more than 2 years from data gathering were included.

b Results with statistical significance.

**Table 6:** Change in PROM scores at 3-, 12- and 24-months’ follow-up

Follow-up Trochlear wear group	Mean (SD)	Estimated change	
		Crude (CI)	Adjusted (CI)
OKS at 3 months (n = 395)			
0	10.0 (8.5)		
1–2	11.3 (9.0)	1.3 (–0.66 to 3.2)	1.1 (–0.60 to 2.8)
3–4	10.4 (8.4)	0.34 (–2.2 to 2.9)	–0.65 (–2.9 to 1.6)
OKS at 12 months (n = 378)			
0	14.1 (8.9)		
1–2	15.4 (8.4)	1.3 (–0.65 to 3.3)	0.78 (–0.96 to 2.5)
3–4	13.7 (9.2)	–0.34 (–3.0 to 2.3)	–0.60 (–3.0 to 1.8)
OKS at 24 months (n = 284)			
0	14.2 (8.9)		
1–2	16.3 (8.1)	2.0 (–0.19 to 4.3)	2.0 (0.05 to 4.0) **^a^**
3–4	13.8 (10.2)	–0.47 (–3.5 to 2.6)	–0.66 (–3.4 to 2.1)
FJS at 3 months (n = 395)			
0	33.6 (26.2)		
1–2	37.9 (26.3)	4.3 (–1.3 to 10)	4.4 (–1.1 to 9.8)
3–4	33.7 (23.2)	0.11 (–7.5 to 7.7)	0.02 (–7.2 to 7.3)
FJS at 12 months (n = 378)			
0	44.8 (28.8)		
1–2	46.8 (27.4)	2.1 (–4.2 to 8.3)	1.62 (–4.3 to 7.5)
3–4	37.9 (26.8)	–7.1 (–16 to 1.4)	–6.6 (–15 to 1.4)
FJS at 24 months (n = 284)			
0	44.6 (29.1)		
1–2	50.2 (27.2)	5.6 (–1.7 to 13)	6.3 (–0.62 to 13)
3–4	39.7 (29.9)	–4.9 (–15 to 5.0)	–3.0 (–13 to 6.5)
APQ at 3 months (n = 395)			
0	31.7 (29.5)	–	
1–2	38.6 (31.3)	7.0 (0.35 to 14) **^a^**	8.5 (2.1 to 15) **^a^**
3–4	28.0 (29.9)	–3.9 (–13 to 5.1)	–1.8 (–10 to 6.7)
APQ at 12 months (n = 378)			
0	46.1 (34.5)	–	
1–2	49.5 (32.5)	3.2 (–4.3 to 11)	4.10(–3.0 to 11)
3–4	40.9 (35.8)	–4.1 (–14 to 6.3)	–0.23 (–9.8 to 9.4)
APQ at 24 months (n = 284)			
0	44.7 (37.2)	–	
1–2	53.0 (32.4)	8.4 (–0.71 to 18)	11 (2.2 to 19) **^a^**
3–4	42.6 (39.9)	–2.1 (–15 to 10)	2.4(–9.3 to 14)

For abbreviations, see Table 1.To calculate the changes, preoperative score was subtracted at each subsequent follow-up.

Multiple linear regression models were used for analysis, showing both the crude estimates and estimates adjusted for sex, BMI, age, and preoperative score.

a Results with statistical significance.

Between 78% and 79% achieved PASS-OKS and between 77% and 80% achieved MIC-OKS out of 549 patients 12 months after surgery across the 5 imputations. While no statistically significant differences in achieving PASS and MIC were observed between groups, 67–73% of patients in group 3–4 achieved PASS compared with 77–80% in group 0 (OR 0.66, CI 0.32–1.4). In group 3–4, 66–74% achieved MIC compared with 75–81% in group 0 (OR 0.57, CI 0.25–1.3) (Table 7). Similar results were found in the complete-case non-imputed cohort (Table 8, see Appendix)

**Table 7:** Patients who achieved patient acceptable symptom state and minimal important change after 12 months

Trochlear wear group	Total n	n (%)	PASS-OKS OR (CI)	Adjusted OR (CI)	n (%)	MIC-OKS OR (CI)	Adjusted OR (CI)
0	200–204	155–163 (77–80)	-	-	152–161 (75–81)	-	-
1–2	256–262	203–217 (79–83)	1.2 (0.66–2.1)	1.2 (0.67–2.3)	204–215 (79–82)	1.2 (0.65–2.4)	1.1 (0.58–2.0)
3–4	85–91	60–63 (67–73)	0.63 (0.31–1.3)	0.66 (0.32–1.4)	59–67 (66–74)	0.67 (0.30–1.5)	0.57 (0.25–1.3)

PASS-OKS = patient acceptable symptom state for the Oxford Knee Score. MIC-OKS = minimal important change for the Oxford Knee Score. OR = odds ratio. CI = confidence interval. The results are presented as the minimum and maximum number and percentage of patients achieving PASS and MIC based on pooled data from 5 imputed datasets. Results are pooled multiple logistic regression models applied to 5 imputed datasets, showing both the crude estimates and estimates adjusted for sex, BMI, age, and preoperative score. Trochlear wear 0 was used as reference group.

**Table 8:** Patients who achieved patient acceptable symptom state (PASS) and minimal important change (MIC) after 12 months in complete-case (non-imputed) cohort

Trochlear wear group	n (%)	PASS-OKS OR (CI)	Adjusted OR (CI)	n (%)	MIC-OKS OR (CI)	Adjusted OR (CI)
0	110 (83)			105 (80)		
1–2	154 (83)	0.96 (0.52–1.7)	1.1 (0.60–2.1)	151 (81)	1.1 (0.63–1.9)	1.0 (0.57–1.9)
3–4	45 (75)	0.60 (0.29–1.3)	0.70 (0.32–1.6)	44 (73)	0.71 (0.35–1.5)	0.65 (0.31–1.4)

For abbreviations, see Table 7.

## Discussion

We aimed to examine the association between PFOA severity and PROM-score changes after mUKA. We found that PROM score improvements were not lower in patients with full-thickness cartilage loss (group 3–4) or in patients with minor cartilage changes (group 1–2) compared with patients with no PFOA (group 0). The largest estimated difference in OKS improvement was not higher than the MIC of 7.1 used in this study or the minimally clinical important difference (MCID) of 5 used in other literature [23]. For FJS and APQ, we did not find clinically relevant differences either.

Our results are supported by other studies [9-12] also showing no difference in PROM or in function between knees with exposed bone at the PFJ and those without exposed bone. However, it is worth noting that these studies may not be entirely comparable, due to different follow up periods. Berger et al. [12] collected data with a mean follow-up of 39 ± 25 months, while our study uniformly collected data at 3, 12, and 24 months.

In addition to examining the PROM development, we have also looked at the proportion of patients who have achieved both PASS and MIC across the groups, and no statistically significant results were found. Group 3–4, defined as patients with cartilage changes of > 50 % of depth or to the bone, tended to be less likely to achieve both PASS-OKS and MIC-OKS. However, with the potential of 73–74% achieving PASS and MIC in Group 3–4 compared with 75–79% in Group 0 and 1–2, the clinical relevance is likely negligible.

It has been suggested that chondral lesions, in the PFJ, have no influence on the postoperative function of UKA, because most people with PFAO are asymptomatic [24]. Noble and Hamblen reported in an article from 1975 an 85% incidence rate of PFOA in a study of 100 randomly selected corpses without any anterior knee pain and with an average age of 65 years [25]. Horga et al. [26] conclude in a study from 2020 that nearly all knees of asymptomatic adults had abnormalities on MRI, among 57% showing cartilage abnormalities at the PFJ. It is therefore likely that PFOA is asymptomatic in many elderly people, including patients eligible for UKA surgery [27-29].

Our study indicated that mUKA patients with PFOA did not have worse outcomes compared with mUKA patients without. Paradoxically, minor PFOA (group 1–2) was associated with greater improvements in APQ and OKS scores at follow-ups. A similar paradox has previously been described [9,10]. Beard et al. found in 2 different studies that patients with full-thickness cartilage loss in the PFJ tended to have better development in PROM scores after mUKA than those without. The reasons for these results are likely multifactorial and require further study.

### Limitations

Although we included a large number of patients, our study has some limitations. We lack information on the PROM scores in some patients with incomplete forms but had high follow-up rates and used imputation to address the missing data. Furthermore, patients who underwent UKA on both knees were seen as 2 separate cases and this could influence the risk of bias as the results may be influenced by each other. The sample was not equally divided among the groups as only 16% had severe/full cartilage loss (group 3–4). Despite the uneven distribution, the characteristics compared across the groups are similar. Furthermore, the patellofemoral cartilage changes were assessed intraoperatively, instead of using radiographs, relying on the subjective assessments of the surgeons.

Additionally, we recognize the limitation of not using PASS and MIC thresholds for all questionnaires. Our decision to use locally defined MIC and PASS values was made to ensure that the clinical relevance was based on data directly applicable to our cohort. While ensuring internal validity, this might limit comparability with studies investigating PASS and MIC.

### Conclusion

We found that cartilage wear in the trochlea was not associated with reduced PROM scores following mobile bearing mUKA. Moreover, when comparing the potential to achieve PASS and MIC, we found no clear clinically relevant differences. The results support that PFOA, excluding severe lateral facet PFOA, should not be considered a contraindication to performing mUKA.

## Notes

JWO and CBJ had full access to all data in the study and all authors take responsibility for the integrity of the data and the accuracy of the data analysis. Study concept and design: CBJ and AT. Acquisition, analysis, and interpretation of data: all authors. Drafting of the manuscript: JWO, CBJ, and KIB. Critical revision of the manuscript: all authors.

Handling co-editors: Keijo Mäkelä and Robin Christensen

*Acta* thanks Margareta Hedström and other anonymous reviewers for help with peer review of this manuscript.
